# Observational study evaluating the effectiveness of physician‐targeted education for improving glycemic management of patients with type 2 diabetes (BEYOND II)†

**DOI:** 10.1111/1753-0407.12963

**Published:** 2019-08-06

**Authors:** Jianping Weng, Jiajun Zhao, Zhiguang Zhou, Xiaohui Guo, Dajin Zou, Qiuhe Ji, Nanwei Tong, Qifu Li, Jun Zhu, Qiang Li, Guijun Qin, Ping Feng, Liyong Yang, Zhengnan Gao, Lulu Chen, Hong Li, Yiming Li, Longyi Zeng, Dalong Zhu, Juming Lu, Tianhong Luo, Nan Cui

**Affiliations:** ^1^ Department of Endocrinology, The First Affiliated Hospital, Division of Life Sciences and Medicine University of Science and Technology of China Hefei China; ^2^ Department of Endocrinology Shandong Provincial Hospital Affiliated to Shandong University Jinan China; ^3^ Institute of Metabolism and Endocrinology, 2nd Xiangya Hospital, Central South University, Key Laboratory of Diabetes Immunology, Ministry of Education National Clinical Research Center for Metabolic Diseases Changsha China; ^4^ Department of Endocrinology Peking University First Hospital Beijing China; ^5^ Department of Endocrinology, Changhai Hospital The Second Military Medical University Shanghai China; ^6^ Department of Endocrinology, Xijing Hospital The Fourth Military Medical University Xi'an China; ^7^ Division of Endocrinology and Metabolism West China Hospital of Sichuan University Chengdu China; ^8^ Department of Endocrinology The First Affiliated Hospital of Chongqing Medical University Chongqing China; ^9^ The First Affiliated Hospital Xinjiang Medical University Urumqi China; ^10^ The Second Affiliated Hospital of Harbin Medical University Harbin China; ^11^ Division of Endocrinology, Department of Internal Medicine The First Affiliated Hospital of Zhengzhou University Zhengzhou China; ^12^ Department of Endocrinology The General Hospital of Tianjin Medical University Tianjin China; ^13^ Department of Endocrinology The First Affiliated Hospital of Fujian Medical University Fuzhou China; ^14^ Department of Endocrinology Dalian Municipal Central Hospital Affiliated of Dalian Medical University Dalian China; ^15^ Department of Endocrinology, Union Hospital, Tongji Medical College Huazhong University of Science and Technology Wuhan China; ^16^ Department of Endocrinology and Metabolism Zhejiang University Affiliated Sir Run Run Shaw Hospital, School of Medicine Hangzhou China; ^17^ Department of Endocrinology and Metabolism, Huashan Hospital Fudan University Shanghai China; ^18^ Department of Endocrinology The Third Affiliated Hospital of Sun Yat‐sen University Guangzhou China; ^19^ Department of Endocrinology, Nanjing Drum Tower Hospital The Affiliated Hospital of Nanjing University Medical School Nanjin China; ^20^ Department of Endocrinology The General Hospital of the People's Liberation Army Beijing China; ^21^ Sanofi (China) Investment Co. Ltd Shanghai China

**Keywords:** basal insulin, education, glycemic management, physician, type 2 diabetes, 基础胰岛素, 教育, 血糖管理, 医生, 2型糖尿病

## Abstract

**Background:**

Because there has been no quality improvement initiatives targeting patients with type 2 diabetes (T2D) receiving basal insulin therapy, this study evaluated the effectiveness of physician‐targeted education for optimizing glycemic management in these patients in China.

**Methods:**

This multicenter open‐label observational study conducted across China had a baseline sample survey, followed by a 6‐month education program, and ended with a post‐education sample survey. Education based on T2D treatment guidelines was given at Months 1 and 3, and was reinforced by self‐audit every month. Each hospital enrolled 100 patients with T2D receiving basal insulin at both the baseline and post‐education survey. The primary outcome was the proportion of hospitals meeting individual improvement goals. The goal setting was based on the proportion of patients achieving HbA1c <7.0% in each hospital at the time of the baseline survey.

**Results:**

Overall, the individual improvement goal was achieved by 35 centers (49%). Hospitals with poor glycemic management at the baseline survey had higher possibility to improve at post‐education survey. Two large sample surveys at baseline and post‐education showed improved glucose management among these hospitals. A higher proportion of patients achieved HbA1c <7.0% in the post‐education survey (27.2% vs 36.5%; *P* < 0.001) with reduced HbA1c levels (8.10% vs 7.72%; *P* < 0.001). Questionnaires from 723 physicians showed that confidence and practice of basal insulin use were significantly improved.

**Conclusions:**

Physician‐targeted education improved glycemic management of patients with T2D in 71 hospitals in China, and was more effective at hospitals with poor glycemic management at the baseline survey.

## Graphical abstract

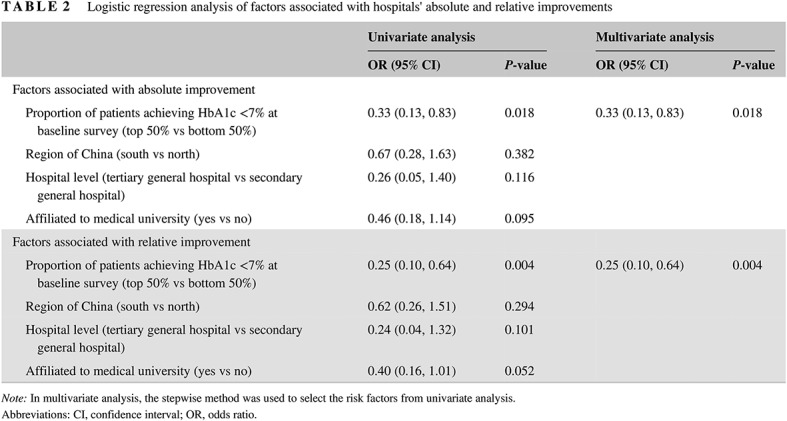

**Highlights**

- This study investigated whether physician‐targeted education can improve outcomes in Chinese patients with type 2 diabetes receiving basal insulin therapy.
- The 6‐month education program improved glycemic management in approximately 50% of participating hospitals. More patients achieved HbA1c < 7% at the time of the post‐education sample survey than at the baseline sample survey.
- Physician‐targeted education was more effective at hospitals that had poor glycemic management at the baseline sample survey.

## 摘要

**背景:**

由于尚无接受基础胰岛素治疗的2型糖尿病(type 2 diabetes, T2D)患者生活质量改善的研究, 本研究评估了以医生为目标的教育对优化中国此类患者血糖管理的有效性。

**方法:**

这是一项在中国进行的多中心、开放式、观察性研究, 研究首先进行了基线的抽样调查, 随后是为期6个月的教育项目以及教育后的抽样调查。基于T2D治疗指南的医生教育项目在第1和第3个月进行, 并通过每月自评加强教育效果。每家医院在基线和教育后调查中均招募了100名接受基础胰岛素治疗的T2D患者。主要结果为达到预设改善目标的医院比例, 目标的设定基于各医院基线调查时糖化血红蛋白(HbA1c)<7.0%的患者比例。

**结果:**

总计35个中心(49%)达到了预设的改善目标。基线调查时血糖管理较差的医院在教育后调查中, 达到目标的可能性更高。基于基线和教育后的两次大样本调查结果显示, 经过以医生为目标的教育, 总体的血糖管理有所改善。在教育后调查中患者HbA1c水平降低(8.10% vs. 7.72%；*P*<0.001), 且HbA1c <7.0%的比例增高(27.2% vs. 36.5%；*P*<0.001)。723名医生的问卷调查显示, 基础胰岛素使用的信心和应用能力显著提高。

**结论:**

在中国的71家医院开展的医生教育提升了他们对于T2D患者的血糖管理水平, 并且基线调查时血糖管理较差的医院提升效果更好。

## 1 INTRODUCTION

A worldwide diabetes epidemic continues to unfold; according to the International Diabetes Federation, in 2017 there were 425 million people affected by diabetes worldwide, with most having type 2 diabetes (T2D).^1^ Due to the progressive nature of T2D, most patients will eventually require insulin therapy.^2^, ^3^ Both international and Chinese treatment guidelines recommend the initiation of basal insulin (BI) for patients unable to achieve glycemic targets with one to two oral antidiabetic drugs (OADs).^4^, ^5^, ^6^

Despite the recommendations of evidence‐based guidelines, large gaps exist globally in the achievement of glycemic control for patients with T2D receiving BI in clinical practice.^7^, ^8^, ^9^ For example, a retrospective analysis using data from a US claims database indicated that the proportion of patients achieving HbA1c <7.0% (53 mmol/mol) was similar for BI users at baseline (26%) and at 3 months follow‐up (27%).^10^ Furthermore, the large Observational Registry of Basal Insulin Treatment (ORBIT) study found that BI was initiated relatively late with mean HbA1c of 9.6% (81 mmol/mol).^11^ Another multicenter cross‐sectional survey conducted in China revealed that of 80 973 patients treated by BI plus OAD(s), only 26.21% achieved HbA1c <7% (53 mmol/mol).^12^ Thus, achieving and maintaining glycemic control in patients receiving BI therapy is a global challenge.

In China, the China Guideline for Type 2 Diabetes is enforced by the Chinese Diabetes Society (CDS) by creating awareness and knowledge exchange.^6^ However, the awareness and implementation of evidence‐based T2D treatment guidelines varies across different geographical regions of China, hospital grades, professional status, and specialities. Reportedly, less than 30% of physicians completely understand the guidelines and apply them in practice.^13^ For several decades, quality improvement interventions directed at patients, doctors, and health systems have aimed to address gaps in the management of T2D not fully addressed through new therapeutics or devices.^14^, ^15^ Results from a large meta‐analysis showed that predefined quality improvement strategies led to improvements in glycemic control.^14^

However, to the best of the authors' knowledge, there have been no previous quality improvement initiatives that focused on patients already receiving BI therapy,^16^, ^17^, ^18^ whose glycemic control is typically relatively poor.^10^, ^11^, ^12^ Furthermore, although both nurses and patients play an important role in quality improvement initiatives, physicians are particularly key in adopting guidelines and improving glycemic control for patients receiving insulin.^19^, ^20^, ^21^ The aim of the BEYOND II study was to evaluate the effectiveness of physician‐targeted education for improving management in T2D patients receiving BI therapy.

## 2 METHODS

### 2.1 Study design

The BEYOND II study was a multicenter open‐label observational study conducted at centers across China from October 2015 to March 2017 (Table S1). The study consisted of a baseline sample survey to evaluate glucose control in the hospital before education, followed by a 6‐month physician‐targeted education program, and ending with a post‐education sample survey to evaluate glucose control in the hospital after education (Figure S1). During both survey periods, physicians at each study site were mandated to consecutively enroll 100 individuals (200 in total) with T2D receiving BI and collect laboratory test results from routine practice. Patients enrolled at the time of the post‐educational survey could be different to those enrolled at the time of the baseline survey. To reduce selection bias, all data were collected within 2 months of recruitment of the first patient at each center and recorded in an electronic case report form (e‐CRF). Participating physicians' confidence and daily practice in BI treatment were also assessed at baseline and after the education intervention using questionnaires.

The study protocol was approved by the Clinical Trial Ethics Committee of the Third Affiliated Hospital, Sun Yat‐sen University (Reference no. [2015] 2‐152 on July 21, 2015). The study was conducted in accordance with the Declaration of Helsinki and in‐line with The International Council for Harmonization of Technical Requirements for Pharmaceuticals for Human Use guidelines for Good Clinical Practice (GCP) and Chinese GCP. Written informed consent was obtained from each study participant. This study is registered in the Chinese Clinical Trials registry (ID: ChiCTR‐OOC‐15006935).

### 2.2 Study center and physician selection criteria

Endocrinology departments at Tier 3 or Tier 2 hospitals across China with a head of department willing to support the implementation of the education and adopt a standard T2D treatment pathway were eligible for inclusion in this study. Most T2D patients in China are treated at Tier 2 and 3 hospitals; therefore, eligible study centers represented the standard of care in China.

Heads of enrolled endocrinology departments conferred with departmental physicians and nominated participants. An inclusion target of ≥60% of outpatient endocrinologists at each study center was set to provide a representative sample of the overall treatment quality. Participating physicians were required to complete the whole study process, and replacement of physicians during the study was not allowed.

### 2.3 Patient inclusion criteria

Adults (age ≥ 18 years) with T2D who had received BI‐based therapy as outpatients for ≥3 months were eligible for inclusion in the study. Study subjects were followed‐up for ≥3 months prior to enrolment at the respective study center, with HbA1c and fasting plasma glucose (FPG) measurements available 1 month before entering the study. Because the present study was an observational study, no medication was provided by the sponsor. The use of OADs and prandial insulin, as well as the BI dose, were chosen at the participating physicians' discretion in line with treatment guidelines and local label indications.

### 2.4 Education and study committee

Physician education was based on a standard T2D treatment pathway and incorporated self‐audit and regular peer‐to‐peer discussion. The treatment pathway followed 2013 CDS^6^ (Figure S2) and 2013 American Association of Clinical Endocrinologists/American College of Endocrinology^22^ (Figure S3) guidelines. Training covered offering advice on diet, smoking cessation, daily physical activity, and maintenance of a healthy weight, as well as information about insulin preparations, correct dosing, when and how to administer insulin, self‐monitoring blood glucose, and management and prevention of hypoglycemia.

Participating physicians attended an initial face‐to‐face interactive training workshop provided by the Study Committee. Participants then applied the standard T2D treatment pathway, insulin initiation and titration scheme, and appropriate patient education in outpatient practice for 6 months. During this 6‐month period, a regular self‐audit regarding implementation of the standard insulin treatment pathway was performed every month. All participating physicians were required to attend the monthly meeting to discuss any issue of BI management during daily practice, share valuable experiences, and come to potential solutions after peer‐to‐peer discussion. The principal investigator of each center was responsible for self‐audit in his or her center.

### 2.5 Objectives and evaluation criteria

The primary endpoint of BEYOND II was the percentage of hospitals meeting individual improvement goals. The goal setting was based on the proportion of patients achieving HbA1c <7.0% (53 mmol/mol) in each hospital at the baseline sample survey. The Study Committee member and principle investigator at each study center discussed the baseline data and decided on an appropriate improvement goal for each center, taking into account relevant factors such as patient characteristics and available resources.^3^

Secondary endpoints included assessment of glycemic control and safety in the baseline and post‐education surveys, as indicated by mean HbA1c and FPG, the proportion of patients achieving HbA1c <7% and FPG <6.1 mM, and the frequency of hypoglycemic events (blood glucose ≤3.9 mM) and severe hypoglycemic events (hypoglycemic episodes requiring the assistance of another person or admission to hospital) in the 2 weeks before enrolment. Physicians' confidence and daily practice in BI treatment were assessed by questionnaire (Figures S4,S5).

Exploratory objectives included investigation of the relationship between hospital characteristics at the baseline survey and absolute and relative improvements in the hospital at the post‐education survey.

### 2.6 Statistical analysis

The primary statistical objective was to estimate the percentage of hospitals meeting individual improvement goals, which is provided with the corresponding 95% confidence intervals (CI). A sample size of 150 Tier 2 and 3 hospitals was calculated to allow estimation of two‐sided 95% CIs for the rate of hospitals that met improvement goals with a precision of approximately ±8.3%, assuming 50% of hospitals would meet improvement goals (the five county‐level hospitals were included in an exploratory group; data from these hospitals will be assessed separately).

A sample size of approximately 100 subjects per cohort in each study center was calculated to allow estimation of the two‐sided 95% CIs for the proportion of subjects achieving HbA1c <7% (53 mmol/mol) with a precision of approximately ±10%, assuming 50% of patients would achieve HbA1c <7% (53 mmol/mol). An HbA1c of <7% (53 mmol/mol) was chosen in the sample size estimation because this was used to set improvement goals for all study centers.

Continuous variables are summarized using descriptive statistics as the mean ± SD or as the median (range). Major continuous variables included mean HbA1c and mean FPG. It was assumed that for the large sample size the data would be normally distributed. A two‐sample *t* test was used to compare baseline and post‐educational (6‐month) data for continuous variables.

Discrete variables are summarized in frequency tables. Major discrete variables included the percentage of patients achieving glucose goal (HbA1c <7%), the percentage of patients with achieving the FPG goal (<6.1 mM), hypoglycemia rate, and severe hypoglycemia rate. Chi‐squared tests were used for comparisons of baseline and post‐educational (6‐month) data.

Univariate and multivariate regression analysis were used to assess factors affecting hospitals' absolute and relative improvements in glycemic management. The factors included in the univariate analysis were the proportion of patients achieving HbA1c <7% at the baseline survey (top 50% vs bottom 50%), region of China (south vs north), hospital level (tertiary general hospital vs secondary general hospital), and affiliated teaching hospital of a medical university (yes vs no). The stepwise method was used to select the risk factors in multivariate analysis.

Values for missing data were not imputed unless stated otherwise.

### 2.7 Data accessibility

Qualified researchers may request access to patient‐level data and related study documents, including the clinical study report, study protocol with any amendments, blank case report form, statistical analysis plan, and dataset specifications. Patient‐level data will be anonymized and study documents will be redacted to protect the privacy of the trial participants. Further details on Sanofi's data sharing criteria, eligible studies, and process for requesting access can be found at https://www.clinicalstudydatarequest.com/ (accessed 08 July 2019).

## 3 RESULTS

### 3.1 Primary endpoint

In all, 73 Tier 2 and 3 hospitals entered the study, and 71 completed the post‐education patient enrolment. Of the 71 hospitals that completed the study, 63 were Tier 3 hospitals and 8 were Tier 2 hospitals; 34 hospitals were located in north China and 37 hospitals were located in south China; 26 were affiliated with a medical university. At the baseline survey, the proportion of patients achieving HbA1c <7% (53 mmol/mol) was <20% at 11 hospitals, 20%‐35% at 47 hospitals, and ≥ 35% at 13 hospitals. At the post‐education survey, the proportion of patients achieving HbA1c <7% (53 mmol/mol) was <20% at 5 hospitals, 20%‐35% at 30 hospitals, and ≥ 35% at 36 hospitals.

The primary endpoint was achieved by 35/71 hospitals (49.3%; 95% CI 37.2%‐61.4%). The number of hospitals with >0% absolute improvement in the proportion of patients achieving HbA1c <7% (53 mmol/mol) was 58/71 (81.7%), of which 41 (70.7%) achieved an improvement of >5% (Table S2). Detailed improvement data for each hospital are provided in Table S3.

### 3.2 Factors related to hospital improvement

In all, 71 hospitals were included in the analysis of the relationship between hospital characteristics at the baseline sample survey and absolute or relative improvement at the post‐education sample survey. The definition of improvement was based on the change in the proportion of patients achieving HbA1c <7% between the baseline and post‐education sample surveys in the participating hospitals. The distribution of hospitals by absolute improvement (no improvement, ≤10% and > 10% improvement) differed significantly between the top 50% and bottom 50% of hospitals stratified by proportion of patients achieving HbA1c <7% at the baseline survey. A similar difference was observed between hospitals affiliated with medical universities and those that were not. In contrast, no difference in distribution was observed according to region or hospital tier (Table 1). Similar results were observed for relative improvement (no improvement, ≤30% and >30% improvement; Table 1).

**Table 1:** Distribution of hospitals by absolute and relative improvement

Hospital characteristics at baseline survey	Hospitals by absolute improvement				Hospitals by relative improvement			
	No improvement (*n* = 13)	≤10% (*n* = 23)	>10% (*n* = 35)	*P*‐value	No improvement (*n* = 13)	≤30% (*n* = 24)	>30% (*n* = 34)	*P*‐value
Proportion of patients achieving HbA1c <7% at baseline survey				0.047				0.013
Top 50%	10 (76.9)	12 (52.2)	13 (37.1)		10 (76.9)	14 (58.3)	11 (32.4)	
Bottom 50%	3 (23.1)	11 (47.8)	22 (62.9)		3 (23.1)	10 (41.7)	23 (67.6)	
Region of China				0.523				0.382
South	7 (53.8)	14 (60.9)	16 (45.7)		7 (53.8)	15 (62.5)	15 (44.1)	
North	6 (46.2)	9 (39.1)	19 (54.3)		6 (46.2)	9 (37.5)	19 (55.9)	
Hospital level				0.222				0.198
Tertiary general hospital	13 (100.0)	21 (91.3)	29 (82.9)		13 (100.0)	22 (91.7)	28 (82.4)	
Secondary general hospital	0	2 (8.7)	6 (17.1)		0	2 (8.3)	6 (17.6)	
Affiliated to medical university				0.002				0.003
Yes	10 (76.9)	4 (17.4)	12 (34.3)		10 (76.9)	5 (20.8)	11 (32.4)	
No	3 (23.1)	19 (82.6)	23 (65.7)		3 (23.1)	19 (79.2)	23 (67.6)	

*Note*: Unless indicated otherwise, data are given as n (%), where n refers to the number of hospitals throughout. *P*‐values were calculated using Chi‐squared tests.

Multivariate analysis revealed that only the variable “hospitals stratified by proportion of patients achieving HbA1c <7% at baseline survey (top 50% vs bottom 50%)” was significantly negatively associated with both absolute improvement (odds ratio [OR] 0.33; 95% CI 0.13‐0.83; *P* = 0.018) and relative improvement (OR 0.25; 95% CI 0.10‐0.64; *P* = 0.004; Table 2). No association was found between hospital region, hospital level, or affiliation status with medical universities.

**Table 2:** Logistic regression analysis of factors associated with hospitals' absolute and relative improvements

	Univariate analysis		Multivariate analysis	
	OR (95% CI)	*P*‐value	OR (95% CI)	*P*‐value
Factors associated with absolute improvement				
Proportion of patients achieving HbA1c <7% at baseline survey (top 50% vs bottom 50%)	0.33 (0.13, 0.83)	0.018	0.33 (0.13, 0.83)	0.018
Region of China (south vs north)	0.67 (0.28, 1.63)	0.382		
Hospital level (tertiary general hospital vs secondary general hospital)	0.26 (0.05, 1.40)	0.116		
Affiliated to medical university (yes vs no)	0.46 (0.18, 1.14)	0.095		
Factors associated with relative improvement				
Proportion of patients achieving HbA1c <7% at baseline survey (top 50% vs bottom 50%)	0.25 (0.10, 0.64)	0.004	0.25 (0.10, 0.64)	0.004
Region of China (south vs north)	0.62 (0.26, 1.51)	0.294		
Hospital level (tertiary general hospital vs secondary general hospital)	0.24 (0.04, 1.32)	0.101		
Affiliated to medical university (yes vs no)	0.40 (0.16, 1.01)	0.052		

*Note*: In multivariate analysis, the stepwise method was used to select the risk factors from univariate analysis.

Abbreviations: CI, confidence interval; OR, odds ratio.

### 3.3 Patient profiles at the baseline and post‐education sample surveys

In all, 6561 patients were enrolled in the baseline sample survey, with 6386 evaluable patients. Following the education program, 6413 patients were enrolled into the post‐education sample survey, with 6353 evaluable patients. Overall, the demographics of patients in the baseline and post‐education surveys were comparable, with a similar mean age, body mass index, duration of T2D, prevalence of diabetic complications, and mean daily BI dose (Table S4).

Overall, in the post‐education sample survey, patients' glycemic control was improved compared with the baseline sample survey (Table 3). Compared with baseline, patients enrolled in the post‐education survey had a lower mean HbA1c level (8.10 ± 1.73% [65 mmol/mol] vs 7.72 ± 1.58% [61 mmol/mol]; *P* < 0.001) and a higher proportion achieved HbA1c <7% (53 mmol/mol; 27.2% vs 36.5%; *P* < 0.001). Similarly, compared with the baseline survey, patients in the post‐education survey had a lower mean FPG (9.10 vs 8.44 mM; *P* < 0.001) and a greater proportion achieved FPG <6.1 mM (15.6% vs 19.6%; *P* < 0.001) and FPG <7.0 mM (29.5% vs 37.2%; *P* < 0.001). Finally, the rate of hypoglycemia was lower in the post‐education survey, although this difference did not reach statistical significance (4.4% vs 3.8%; *P* = 0.077).

**Table 3:** Summary of glucose management at the baseline and post‐education sample surveys

Variable	Baseline sample survey (*n* = 6386)	Post‐education sample survey (*n* = 6353)	Difference (95% CI)	*P*‐value^a^
Mean (± SD) HbA1c (%)	8.10 ± 1.73	7.72 ± 1.58	−0.38 (−0.43, −0.32)	<0.001
HbA1c <7%	1740 (27.2)	2322 (36.5)	9.3% (7.7%, 10.9%)	<0.001
Adjusted HbA1c target^b^	2183 (34.2)	2743 (43.2)	9.0% (7.3%, 10.7%)	<0.001
Mean (± SD) FPG (mM)	9.10 ± 3.58	8.44 ± 3.17	−0.66 (−0.78, 0.54)	<0.001
FPG <6.1 mM	994 (15.6)	1247 (19.6)	4.1% (2.7%, 5.4%)	<0.001
FPG <7.0 mM	1883 (29.5)	2363 (37.2)	7.7% (6.1%, 9.3%)	<0.001
Incidence of hypoglycemia	282 (4.4)	241 (3.8)	−0.6% (−1.3%, 0.1%)	0.077

*Note*: Unless indicated otherwise, data are given as *n* (%).

Abbreviations: CI, confidence interval; FPG, fasting plasma glucose.

a Chi‐squared test for categorical variables, Student's *t* test for continuous variables.

b Adjusted HbA1c target calculated using an adjusted HbA1c target of ≤7.5% (58 mmol/mol) for patients with existing cardiovascular disease or age ≥ 65 years.

At hospitals that met individualized improvement targets, compared with baseline, the proportion of patients in the post‐education survey achieving HbA1c <7% (53 mmol/mol) was significantly higher (25.6% vs 43.1%; *P* < 0.001), whereas mean HbA1c (8.10% [65 mmol/mol] vs 7.46% [58 mmol/mol]; *P* < 0.001) and FPG (9.02 vs 8.04 mM; *P* < 0.001) levels were significantly lower (Table S5). In contrast, at hospitals not meeting targets, the proportion of patients in the baseline and post‐education surveys achieving HbA1c <7% (53 mmol/mol) was similar (29.1% vs 29.8%; *P* = 0.537), and differences in mean HbA1c (8.08 [61 mmol/mol] vs 7.99% [64 mmol/mol]; *P* = 0.045) and FPG (9.15 vs 8.85 mM; *P* < 0.001) levels were lower in magnitude.

### 3.4 Physician questionnaire analysis

**Figure 1:**
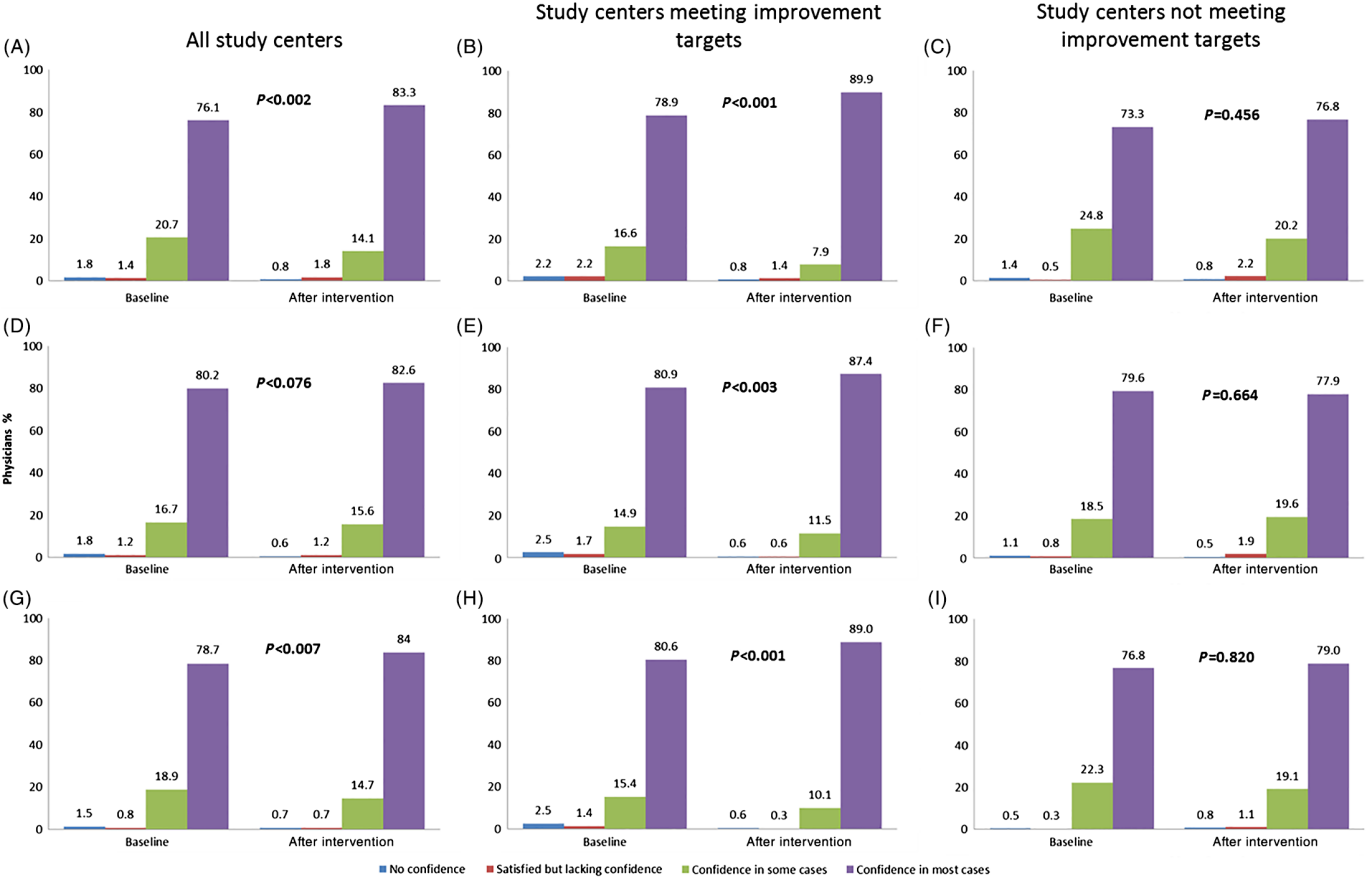
Results of physician (*n* = 723) confidence in the use of basal insulin, assessed by questionnaires, for all study centers, those that achieved individualized improvement targets (*n* = 356), and those that did not (*n* = 367) at baseline and after the 6‐month education intervention. A‐C, Initiation of basal insulin; D‐F, titration of insulin dose to meet fasting plasma glucose (FPG) targets; G‐I, management of hypoglycemia

In all, 793 physicians were included in the study at baseline, of whom 764 took part in the 6‐month education intervention and 723 had evaluable data. According to the baseline survey, 550 (76.1%) physicians self‐reported “confidence in most cases” in initiating BI therapy; this number increased to 602 (83.3%; *P* = 0.002) at the post‐education survey (Figure 1A). Similarly, the number of physicians reporting ‘confidence in most cases’ in management of hypoglycemia also increased between the baseline and post‐education surveys (569 [78.7%] vs 607 [84.0%]; *P* = 0.007; Figure 1G). However, there was no significant difference in the proportion of physicians “confident in most cases” about reaching FPG goals via BI titration between the baseline and post‐education surveys (80.2% vs 82.6%; *P* = 0.076; Figure 1D). Subgroup analysis revealed that study centers meeting individualized improvement targets had significant changes in treatment confidence (Figure 1B,E,H), compared with no significant changes in centers not meeting the improvement targets (Figure 1C,F,I).

**Figure 2:**
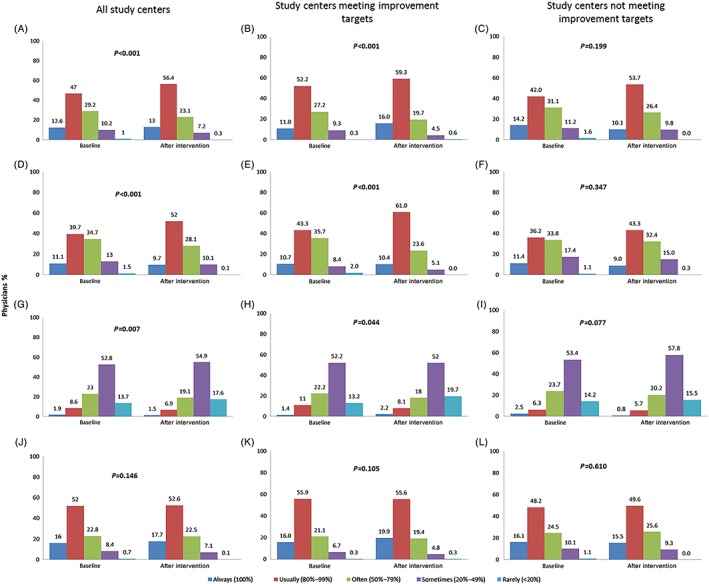
Physician (*n* = 723) clinical practice in the use of basal insulin, assessed by questionnaires, for all study centers, for those that achieved individualized improvement targets (*n* = 356), and those that did not (*n* = 367). A‐C, Use basal insulin to initiate insulin treatment; D‐F, titrate basal insulin using fasting plasma glucose (FPG) <6.1 mM; G‐I, use premixed insulin to replace basal insulin; J‐L, use basal insulin for individuals who do not achieve HbA1c and FPG targets

A survey of daily insulin treatment practice revealed that the proportion of physicians who “always” (100% of the time) or “usually” (80%‐99% of the time) prescribed BI as initial treatment and titrated BI dose to achieve FPG <6.1 mM was higher at the post‐education than baseline survey (Figure 2A,D). Furthermore, the proportion of physicians who ‘always’ or ‘usually’ replaced BI with premixed insulin showed a small decrease after education compared with baseline (Figure 2G). The initiation of BI for individuals not achieving HbA1c and FPG targets was largely comparable at baseline and after the education intervention (Figure 2J). In addition, physicians at study centers that met improvement targets showed more marked changes in clinical practice in terms of BI use following the education intervention (Figure 2B,E,H,K). At centers not meeting targets there was no significant change in clinical practice (Figure 2C,F,I,L).

## 4 DISCUSSION

There is currently a global need to improve rates of glycemic control among patients with T2D receiving BI‐based treatment.^10^, ^11^, ^12^ To the best of the authors' knowledge, BEYOND II is the first study to demonstrate the effectiveness of physician‐targeted education for improving glycemic management of patients with T2D receiving BI. The primary endpoint revealed that the 6‐month education program led to achievement of individualized improvement goals at approximately 50% of hospitals. The primary endpoint was further supported by the finding that the proportion of patients achieving HbA1c <7% at the post‐education sample survey was higher than at the baseline sample survey. Furthermore, multivariate analysis revealed that hospitals with poor glycemic control at the baseline survey had a higher possibility of improving after the 6‐month education intervention; these results indicate that physician‐targeted education may be more effective at hospitals with poor glycemic management at the baseline survey.

The primary objective of this study was to observe changes in glucose management by physicians and hospitals after education. Two sample surveys were the best strategy to meet this objective. During the 6‐month education period, physicians applied the standard treatment procedures recommended in training, which may have improved glucose management and benefitted all the patients treated by them. Following‐up the same 100 patients would have only yielded data on changes in HbA1c in these patients. To ensure all the enrolled patients were affected by the education intervention, only patients who were being followed‐up at the study at site and receiving BI therapy for ≥3 months were included in the study. Thus, the patients received treatment from the trained physicians for at least 3 months before the post‐education survey.

One unique strength of the BEYOND II study is using individualized improvement goals as the primary endpoint. The use of individualized goals provides physicians with a clear overview of glycemic control at their hospital, and gives them tangible improvement goals to achieve. In contrast, all previous quality improvement studies used HbA1c reductions in overall patients as the primary endpoint.^10^, ^11^, ^12^, ^14^, ^18^ Another important strength of this study was the incorporation of multiple elements in the physician‐targeted education, including the use of evidence‐based guidelines and training on how to educate patients, both of which have been shown to be effective in improving diabetes care.^14^, ^18^ Moreover, the implementation of regular peer‐to‐peer review and discussion are suggested as particularly useful in stimulating changes in physicians' habits.^23^, ^24^, ^25^ Finally, this study included a self‐assessment of physicians' confidence and clinical practice in BI use via the use of a questionnaire. These questionnaires enabled investigation of the relationship between physicians' self‐assessed confidence, their behavior in real clinical practice, and the outcomes of diabetes care at their hospitals. The final results demonstrated that physicians' confidence and behavior change were positively associated with improvements in glycemic control at their hospitals.

However, the BEYOND II study may have limitations. First, the lack of a control group did not allow direct comparison of the education vs no education or an alternative education. Second, two separate groups of patients were enrolled at the baseline and post‐education surveys, which may have resulted in selection bias. To reduce the selection bias of the two surveys, a consecutive 2‐month enrollment was adopted. A longer enrolment period (3‐5 months) would have given physicians a chance to select patients with better glycemic control to meet their post‐education target. However, we acknowledge that the selection bias could not be totally avoided in this study. Third, the post‐education survey was conducted immediately after the 6‐month education intervention. We acknowledge that conducting another 2‐month survey 1 year after the completion of the education intervention would have enable us to demonstrate whether the effects of the education intervention are sustainable. Finally, the findings could have been affected by the Hawthorne effect because the physicians were aware of being under observation.^26^ However, a previous study reported limited influence of the Hawthorne effect on patient‐physician visits, except for the subgroup of vulnerable patients, where it slightly affected the observations.^27^

In conclusion, physician‐targeted education improved glycemic management of Chinese patients with T2D in 71 hospitals across China and appeared to be more effective at hospitals with poor mean glycemic control at baseline. However, future studies are warranted to confirm the program's effectiveness (eg, using control groups) and to establish the effectiveness of physicians' education in the whole country.

## DISCLOSURE

All authors declare that sponsorship of this study, including data collection, physicians' training and article processing charges, were funded by Sanofi, Shanghai, China. There are no other relationships or activities that could appear to have influenced the submitted work.

## Supporting information


**Figure S1.** Study schematic.
**Figure S2.** Treatment pathway for type 2 diabetes.
**Figure S3.** American Association of Clinical Endocrinologists basal insulin titration algorithm.
**Figure S4.** Questionnaire used to assess investigators' confidence in basal insulin treatment and daily practice in basal insulin treatment.
**Figure S5.** Questionnaire used to assess investigators' daily practice in basal insulin treatment.
**Table S1.** Hospital and patient distribution in 10 Chinese regions.
**Table S2.** Absolute improvement in the proportion of patients achieving HbA1c <7% (53 mmol/mol) at hospitals that did and did not achieve improvement targets.
**Table S3.** HbA1c target achievement by hospital.
**Table S4.** Patient profile at the baseline and post‐education sample surveys.
**Table S5.** Summary of glycemic management by hospitals achieving or not achieving improvement targets.
