# Supplementary material for: Observational study evaluating the effectiveness of physician‐targeted education for improving glycemic management of patients with type 2 diabetes (BEYOND II)
Source: J Diabetes. 2019 Aug 6;12(1):66–76. doi: 10.1111/1753-0407.12963 (PMC6972623; doi:10.1111/1753-0407.12963)
Supplement: Supplementary file 1 — Figure S1. Study schematic. Figure S2. Treatment pathway for type 2 diabetes. Figure S3. American Association of Clinical Endocrinologists basal insulin titration algorithm. Figure S4. Questionnaire used to assess investigators' confidence in basal insulin treatment and daily practice in basal insulin treatment. Figure S5. Questionnaire used to assess investigators' daily practice in basal insulin treatment. Table S1. Hospital and patient distribution in 10 Chinese regions. Table S2. Absolute improvement in the proportion of patients achieving HbA1c <7% (53 mmol/mol) at hospitals that did and did not achieve improvement targets. Table S3. HbA1c target achievement by hospital. Table S4. Patient profile at the baseline and post‐education sample surveys. Table S5. Summary of glycemic management by hospitals achieving or not achieving improvement targets. [file JDB-12-66-s001.docx]

**An observational study to evaluate the effectiveness of physician-targeted education for improving glycemic management of patients with type 2 diabetes mellitus (BEYOND II)**

# **Supplementary Tables**

**Supplementary Table 1**. Hospital and patient distribution in ten Chinese regions

| Region | | Sample size | Centers |
| --- | --- | --- | --- |
| 1 | Shaanxi, Gansu, Qinghai, Ningxia, Xingjiang | 1,200 | 6 |
| 2 | Liaoning, Jiling, Heilongjiang | 1,200 | 6 |
| 3 | Fujian, Jiangxi, Guangxi, Guangdong, Hainan | 2,600 | 13 |
| 4 | Jiangsu, Zhejiang, Anhui | 2,000 | 10 |
| 5 | Shanghai | 600 | 3 |
| 6 | Beijing | 600 | 3 |
| 7 | Tianjing, Hebei, Shanxi, Inner Mongolia | 2,400 | 12 |
| 8 | Hubei, Hunan | 1,200 | 6 |
| 9 | Henan, Shandong | 1,600 | 8 |
| 10 | Guizhou, Sichuan, Chongqing, Yunnan | 1,600 | 8 |
| **Total** | | **15,000** | **75** |

**Supplementary Table 2.** Absolute improvement in the proportion of patients achieving HbA1c <7% (53 mmol/mol) at hospitals which did and did not achieve improvement targets

|  | Hospitals achieving improvement targets (n=35) | Hospitals not achieving improvement targets (n=36) |
| --- | --- | --- |
| Proportion of patients achieving HbA1c <7%, n (%) |  |  |
| ≤0% | 0 | 13 (36.1) |
| 0%–≤5% | 0 | 17 (47.2) |
| 5%–≤10% | 5 (14.3) | 1 (2.8) |
| 10%–≤15% | 9 (25.7) | 5 (13.9) |
| >15% | 21 (60.0) | 0 |

**Supplementary Table 3.** HbA1c target achievement by hospital

| **Hospital Name** | **Baseline sample survey** | | **Post-education sample survey** | | **Improvement target, %** | **Improvement target achieved (Yes/No)** |
| --- | --- | --- | --- | --- | --- | --- |
|  | **Evaluable patients, n** | **HbA1c <7%, n (%)** | **Evaluable patients, n** | **HbA1c <7%, n (%)** |  |  |
| The First Hospital of Lanzhou University | 56 | 19 (33.9) | 99 | 32 (32.3) | 39 | No |
| The First Affiliated Hospital of Xi’An Jiaotong University | 22 | 6 (27.3) | 16 | 3 (18.8) | 32 | No |
| Qinghai People’s Hospital | 100 | 33 (33.0) | 100 | 24 (24.0) | 50 | No |
| The First Affiliated Hospital of Xinjiang Medical University | 94 | 19 (20.2) | 100 | 37 (37.0) | 31 | Yes |
| The Xinjiang Uygur Autonomous Region People’s Hospital | 99 | 17 (17.2) | 100 | 22 (22.0) | 27 | No |
| The Fifth Affiliated Hospital of Xinjiang Medical University | 98 | 28 (28.6) | 100 | 35 (35.0) | 33 | Yes |
| The General Hospital of Shenyang | 100 | 35 (35.0) | 99 | 36 (36.4) | 40 | No |
| Dalian Central Hospital | 98 | 11 (11.2) | 100 | 45 (45.0) | 30 | Yes |
| Panjin Central Hospital | 100 | 25 (25.0) | 100 | 66 (66.0) | 37 | Yes |
| Shuangshan Hospital of Anshan City | 100 | 24 (24.0) | 100 | 48 (48.0) | 35 | Yes |
| Traditional Chinese Medical Hospital of Jiamusi City | 100 | 37 (37.0) | 99 | 33 (33.3) | 42 | No |
| Heilongjiang Provincial Hospital | 99 | 16 (16.2) | 100 | 46 (46.0) | 30 | Yes |
| Zhuhai People’s Hospital | 94 | 30 (31.9) | 100 | 55 (55.0) | 37 | Yes |
| The Third Affiliated Hospital, Sun Yat-Sen University | 92 | 28 (30.4) | 100 | 48 (48.0) | 45 | Yes |
| The First Affiliated Hospital of Guangzhou Uni. Of Traditional Chinese Medicine | 34 | 8 (23.5) | 47 | 18 (38.3) | 35 | Yes |
| Dongguan People’s Hospital | 99 | 30 (30.3) | 99 | 46 (46.5) | 40 | Yes |
| Guangzhou First People's Hospital | 96 | 23 (24.0) | 100 | 24 (24.0) | 35 | No |
| Shantou Central Hospital | 56 | 10 (17.9) | 74 | 7 (9.5) | 25 | No |
| Guangzhou Hospital Of TCM | 66 | 6 (9.1) | 100 | 10 (10.0) | 15 | No |
| Zhongshan People’s Hospital | 99 | 22 (22.2) | 99 | 24 (24.2) | 32 | No |
| Zhujiang Hospital of South Medical University | 37 | 9 (24.3) | 17 | 4 (23.5) | 32 | No |
| Jiangxi Pingxiang People’s Hospital | 96 | 86 (89.6) | 100 | 60 (60.0) | 90 | No |
| Zhongshan Hospital Xiamen University | 100 | 15 (15.0) | 99 | 34 (34.3) | 20 | Yes |
| The First Affiliated Hospital Of Fujian Medical University | 98 | 30 (30.6) | 98 | 29 (29.6) | 40 | No |
| Fuzhou Second Hospital | 93 | 17 (18.3) | 98 | 18 (18.4) | 25 | No |
| The People’s Hospital of Ruian | 98 | 18 (18.4) | 100 | 32 (32.0) | 35 | No |
| Hangzhou First People’s Hospital | 90 | 32 (35.6) | 76 | 30 (39.5) | 40 | No |
| Shaoxing People’s Hospital | 97 | 26 (26.8) | 98 | 29 (29.6) | 35 | No |
| Yangzhou First People’s Hospital | 98 | 26 (26.5) | 96 | 37 (38.5) | 35 | Yes |
| Nanjing Drum Tower Hospital | 65 | 13 (20.0) | 80 | 28 (35.0) | 30 | Yes |
| The First People’s Hospital of Kunshan | 99 | 21 (21.2) | 72 | 24 (33.3) | 40 | No |
| Changzhou No.2 People’s Hospital | 97 | 20 (20.6) | 100 | 44 (44.0) | 40 | Yes |
| Nanjing General Hospital | 62 | 20 (32.3) | 99 | 33 (33.3) | 37 | No |
| The Second Hospital of Anhui Medical University | 99 | 43 (43.4) | 99 | 53 (53.5) | 55 | No |
| Shanghai Tenth People’s Hospital | 99 | 30 (30.3) | 100 | 34 (34.0) | 38 | No |
| Huashan Hospital Fudan University | 96 | 20 (20.8) | 96 | 23 (24.0) | 25 | No |
| Shanghai Xuhui District Central Hospital | 98 | 27 (27.6) | 99 | 29 (29.3) | 32 | No |
| Beijing Haidian Hospital | 94 | 24 (25.5) | 97 | 35 (36.1) | 35 | Yes |
| Beijing Pinggu Hospital | 96 | 20 (20.8) | 100 | 36 (36.0) | 30 | Yes |
| Peking University First Hospital | 77 | 13 (16.9) | 89 | 27 (30.3) | 22.3 | Yes |
| General Hospital of Tianjin Medical University | 99 | 35 (35.4) | 99 | 55 (55.6) | 40 | Yes |
| Tianjin Nankai Chinese Medicine Hospital | 100 | 35 (35.0) | 100 | 51 (51.0) | 40 | Yes |
| Tianjin First Center Hospital | 96 | 22 (22.9) | 99 | 38 (38.4) | 33 | Yes |
| Xishan Coal Electricity Group Worker General Hospital | 100 | 24 (24.0) | 100 | 34 (34.0) | 31 | Yes |
| Taiyuan People’s Hospital | 100 | 24 (24.0) | 100 | 55 (55.0) | 34 | Yes |
| The First Hospital of Shanxi Medical University | 98 | 23 (23.5) | 100 | 27 (27.0) | 28 | No |
| The Second Hospital Shijiazhuang City | 98 | 23 (23.5) | 100 | 37 (37.0) | 35 | Yes |
| The First Hospital Shijiazhuang City | 98 | 21 (21.4) | 100 | 32 (32.0) | 35 | No |
| Fourth Hospital of Hebei Medical University | 100 | 43 (43.0) | 100 | 37 (37.0) | 48 | No |
| Tangshan Gongren Hospital | 99 | 22 (22.2) | 95 | 24 (25.3) | 30 | No |
| First Hospital of Qinhuangdao | 100 | 40 (40.0) | 100 | 48 (48.0) | 48 | Yes |
| Baoding First Central Hospital | 98 | 25 (25.5) | 100 | 42 (42.0) | 38 | Yes |
| The Third Hospital of Changsha | 99 | 23 (23.2) | 100 | 39 (39.0) | 28 | Yes |
| Xiangya Hospital Central South University | 42 | 21 (50.0) | 24 | 14 (58.3) | 50 | Yes |
| Hunan Provincial People’s Hospital | 2 | 1 (50.0) |  |  | NA | NA |
| The People’s Hospital of Shiyan | 48 | 13 (27.1) | 99 | 46 (46.5) | 37 | Yes |
| The Puai Hospital of Wuhan | 100 | 15 (15.0) | 100 | 27 (27.0) | 30 | No |
| The Third Hospital of Wuhan | 98 | 28 (28.6) | 99 | 32 (32.3) | 34 | No |
| Qingdao Eighth People’s Hospital | 99 | 9 (9.1) | 100 | 22 (22.0) | 22 | Yes |
| Qingdao Endocrine and Diabetes Hospital | 100 | 33 (33.0) | 14 | 9 (64.3) | 39 | Yes |
| Taian City Central Hospital | 98 | 32 (32.7) | 100 | 49 (49.0) | 42 | Yes |
| Shandong Province Traffic Hospital | 99 | 21 (21.2) | 98 | 27 (27.6) | 30 | No |
| Henan Province People’s Hospital | 60 | 15 (25.0) | 78 | 23 (29.5) | 35 | No |
| The First Affiliated Hospital of Henan University of Science And Technology | 98 | 34 (34.7) | 99 | 31 (31.3) | 45 | No |
| Huaihe Hospital of Henan University | 98 | 37 (37.8) | 100 | 49 (49.0) | 42 | Yes |
| West China Hospital Sichuan University | 65 | 19 (29.2) | 18 | 5 (27.8) | 35 | No |
| The First People’s Hospital of Longquanyi District, Chengdu | 100 | 26 (26.0) | 100 | 32 (32.0) | 32 | Yes |
| Chengdu Second People’s Hospital | 74 | 30 (40.5) | 84 | 36 (42.9) | 45 | No |
| The Third People’s Hospital of Chengdu | 84 | 17 (20.2) |  |  | 25 | NA |
| The First Affiliated Hospital of Chongqing Medical University | 100 | 21 (21.0) | 98 | 47 (48.0) | 31 | Yes |
| Xinqiao Hospital, Third Military Medical University | 62 | 24 (38.7) | 32 | 8 (25.0) | 45 | No |
| The Second Affiliated Hospital of Guizhou Medical University | 100 | 29 (29.0) | 100 | 43 (43.0) | 36 | Yes |
| First Affiliated Hospital of Kunming Medical University | 83 | 18 (21.7) | 72 | 5 (6.9) | 25 | No |

**Supplementary Table 4.** Patient profile at baseline and post-education sample survey

| **Variable^a^** | **Baseline survey**  **(n=6386)** | **Post-education survey (n=6353)** | **P-value^b^** |
| --- | --- | --- | --- |
| Age, years | 59.7 (11.95) | 59.7 (11.79) | 0.777 |
| Males, % (n) | 52.9 (3377) | 52.8 (3357) | 0.964 |
| BMI, kg/m^2^ | 25.0 (3.29) | 24.8 (3.13) | <0.001 |
| Smokers, % (n) | 27.8 (1777) | 25.6 (1627) | -- |
| Duration of type 2 diabetes mellitus, years | 10.7 (7.27) | 10.6 (7.20) | 0.208 |
| Diabetic complications occurring in >5% of patients, % (n) |  |  |  |
| Diabetic neuropathy | 28.9 (1846) | 27.6 (1751) | 0.092 |
| Peripheral vascular disease | 16.9 (1080) | 14.3 (907) | <0.001 |
| Diabetic nephropathy | 12.8 (815) | 13.6 (867) | 0.140 |
| Diabetic retinopathy | 5.8 (370) | 6.4 (404) | 0.182 |
| Cardiovascular disease | 5.5 (353) | 4.0 (253) | <0.001 |
| Basal insulin dose, IU/kg/day | 0.24 (0.10) | 0.24 (0.10) | 0.542 |
| Basal insulin regimen, % (n) |  |  | <0.001 |
| Long-acting insulin + OAD(s) | 63.4 (4047) | 67.9 (4313) |  |
| Long-acting insulin + fast-acting insulin | 24.1 (1538) | 22.0 (1400) |  |
| Long-acting insulin only | 7.1 (453) | 5.6 (356) |  |
| Basal insulin + OADs, % (n) | 65.9 (4207) | 70.2 (4462) | <0.001 |
| Basal insulin + α-Glucosidase inhibitor | 10.3 (658) | 10.2 (651) | 0.916 |
| Basal insulin + metformin | 9.3 (594) | 9.4 (597) | 0.853 |
| Basal insulin + metformin + α-Glucosidase inhibitor | 8.7 (554) | 10.0 (633) | 0.012 |
| Basal insulin + metformin + sulfonylureas | 4.2 (268) | 5.0 (315) | 0.040 |
| Basal insulin + sulfonylureas | 3.0 (190) | 2.3 (146) | 0.017 |

^a^Values are mean (standard deviation) unless otherwise specified; ^b^Chi-squared test for categorical variables and Student’s t-test for continuous variables. BMI, body mass index; OAD, oral antidiabetic drug.

**Supplementary Table 5.** Summary of glycemic management by hospitals achieving or not achieving improvement targets

|  | **Hospitals achieving**  **improvement targets** | | | **Hospitals not achieving improvement targets** | | |
| --- | --- | --- | --- | --- | --- | --- |
| **Endpoint^a^** | **Baseline survey**  **(n=3210)** | **Post-education survey**  **(n=3240)** | **P-value** | **Baseline survey**  **(n=3090)** | **Post-education survey**  **(n=3113)** | **P-value** |
| HbA1c, % | 8.10 (1.685) | 7.46 (1.403) | <0.001 | 8.08 (1.763) | 7.99 (1.703) | 0.045 |
| HbA1c <7%, % (n) | 25.6  (822) | 43.1 (1395) | <0.001 | 29.1  (900) | 29.8  (927) | 0.573 |
| FPG, mmol/L | 9.02 (3.453) | 8.04 (2.760) | <0.001 | 9.15 (3.680) | 8.85 (3.504) | <0.001 |
| FPG <6.1 mmol/L, % (n) | 15.2  (488) | 20.5  (665) | <0.001 | 16.1  (496) | 18.7 (582) | 0.006 |

^a^Mean (SD) unless otherwise stated. FPG, fasting plasma glucose.

# **Supplementary Figures**

**Supplementary Figure 1.** Study schematic


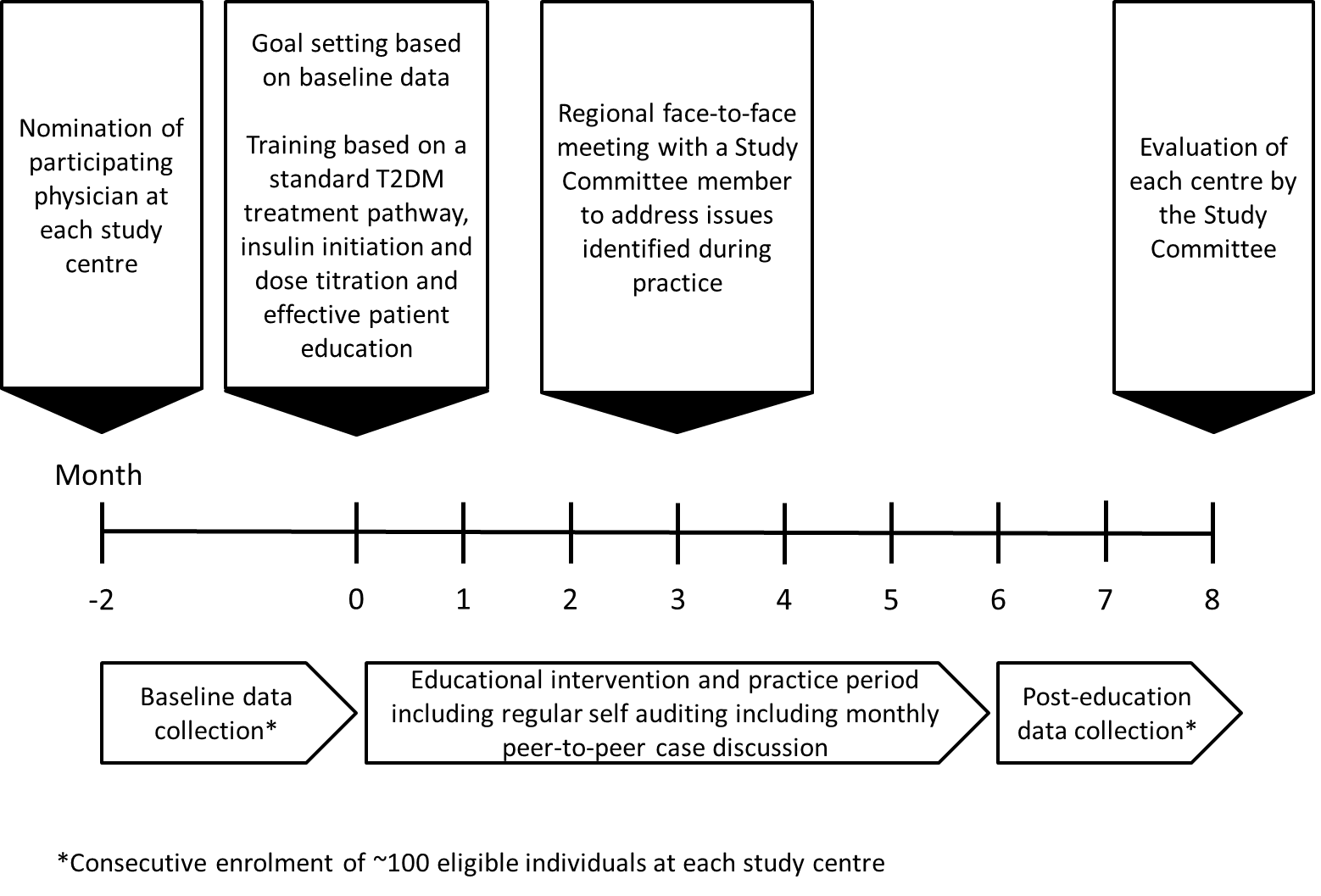


**Supplementary Figure 2.** Treatment pathway for type 2 diabetes mellitus. The BEYOND II study will implement the National Health and Family Planning Commission’s standard basal insulin treatment pathway (2013). The treatment pathway will be supported by physician-specific education on diabetes management and will include regular peer-to-peer discussion and feedback about investigator daily practice.


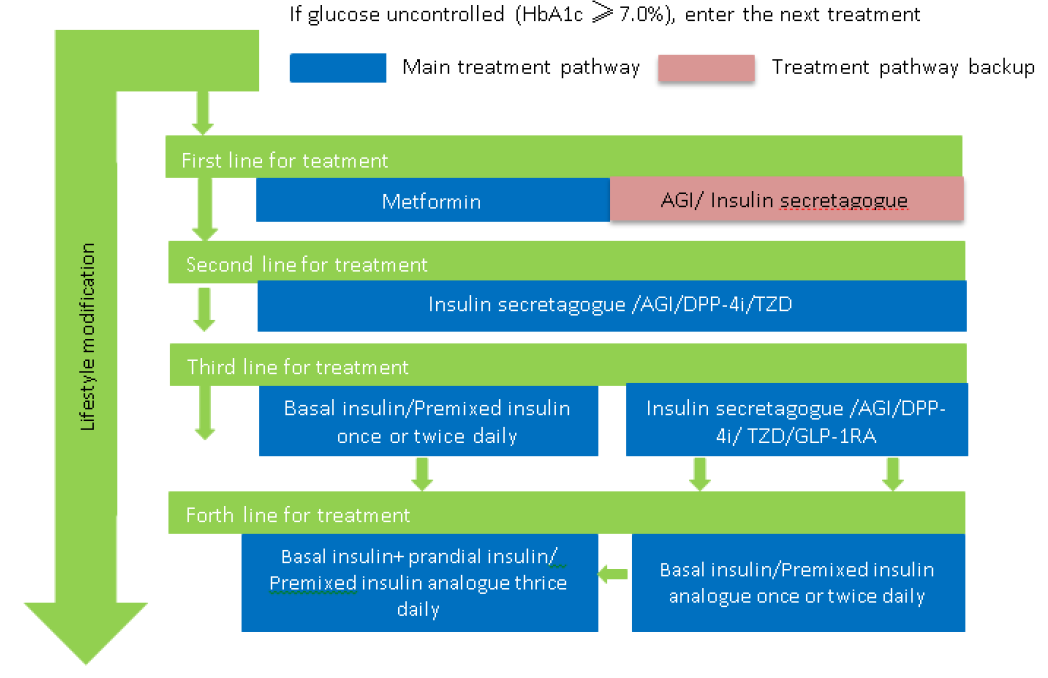


2013 China Guideline for the Treatment of Type 2 Diabetes by Chinese Diabetes Society

**Supplementary Figure 3.** American Association of Clinical Endocrinologists basal insulin titration algorithm, which will be implemented together with the treatment pathway. FBG: fasting blood glucose; HbA1c: glycated hemoglobin; NHP: neutral protamine Hagedorn; TDD: total daily dose.


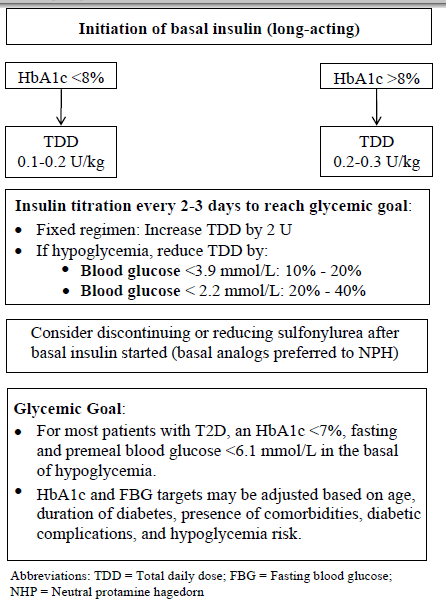


**Supplementary Figure 4.** Questionnaire to assess investigators’ confidence in basal insulin treatment and daily practice in basal insulin treatment

|  | CR1* | CR2* | CR3* | CR4* |
| --- | --- | --- | --- | --- |
| Basal insulin initiation |  |  |  |  |
| Basal insulin titration to achieve fasting blood glucose target |  |  |  |  |
| Hypoglycemia management |  |  |  |  |

*CR1: not confident; CR2: satisfactory but lacking confidence; CR3: confident in some cases; CR4: fully confident in most cases.

**Supplementary Figure 5.** Questionnaire to assess investigators’ daily practice in basal insulin treatment

|  | Always* | Almost always* | Often* | Not very often* | Rarely* |
| --- | --- | --- | --- | --- | --- |
| Use basal insulin as insulin initiation |  |  |  |  |  |
| Titrate basal insulin dose to achieve fasting blood glucose < 6.1 mmol/L |  |  |  |  |  |
| Replace basal insulin with premixed insulin |  |  |  |  |  |
| Use basal insulin in patients whose fasting blood glucose and HbA1c not achieving the goal |  |  |  |  |  |

*Always: 100%; almost always: 80–99%; often: 50–79%; not very often: 20–49%; rarely: less than 20%.
